# Booster vaccination using bivalent DS-5670a/b is safe and immunogenic against SARS-CoV-2 variants in children aged 5–11 years: a phase 2/3, randomized, active-controlled study

**DOI:** 10.3389/fimmu.2024.1445459

**Published:** 2024-09-02

**Authors:** Rino Suzuki, Miharu Suda, Katsuyasu Ishida, Kei Furihata, Aisaku Ota, Kaori Takahashi, Sachiko Sakakibara, Tetsuo Nakayama, Fumihiko Takeshita

**Affiliations:** ^1^ R&D Division, Daiichi Sankyo Co., Ltd., Tokyo, Japan; ^2^ Global DX, Daiichi Sankyo Co., Ltd., Tokyo, Japan; ^3^ Ömura Satoshi Memorial Institute, Kitasato University, Tokyo, Japan

**Keywords:** booster vaccination, children, COVID-19, DS-5670a/b, non-inferiority, omicron variant, SARS-CoV-2

## Abstract

**Background:**

DS-5670 is a messenger ribonucleic acid (mRNA) vaccine platform targeting the receptor-binding domain (RBD) of the spike protein derived from severe acute respiratory syndrome-coronavirus-2 (SARS-CoV-2). Booster vaccination against coronavirus disease 2019 (COVID-19) with monovalent DS-5670a (incorporating mRNA encoding the RBD from the original SARS-CoV-2 strain) or bivalent DS-5670a/b (original and omicron BA.4-5 RBD antigens) is effective and safe in adults. Data from a phase 2/3 active-controlled, non-inferiority, pediatric study evaluating a third booster dose of DS-5670a/b are reported here.

**Methods:**

Children aged 5–11 years who had completed the two-dose primary vaccination series with monovalent BNT162b2 (original strain) at least 3 months prior to enrolment were randomly assigned to receive DS-5670a/b (20 µg of mRNA) or bivalent BNT1 62b2 (original/omicron BA.4-5; 10 µg of mRNA) on Day 1. The primary efficacy endpoint was blood neutralization geometric mean titer (GMT) against SARS-CoV-2 (omicron variant BA.5.2.1) and immune response rate (≥ 4-fold increase in post-vaccination circulating anti-SARS-CoV-2 neutralizing activity) on Day 29.

**Results:**

Among evaluable participants (DS-5670a/b, n = 74; bivalent BNT162b2, n = 75), the adjusted GMT ratio of DS-5670a/b to bivalent BNT162b2 on Day 29 was 1.636 (95% CI, 1.221, 2.190). Immune response rates were ≥ 89% with both study vaccines; adjusted difference 2.6% (95% CI, –7.8, 13.8). The prespecified non-inferiority margins were exceeded, and the study met the primary endpoint. DS-5670a/b also demonstrated broad neutralization activity across recent omicron sublineages and no cases of COVID-19 between Days 8–29 post-administration were reported. There were no novel safety concerns in the pediatric population at data cut-off.

**Conclusions:**

Bivalent DS-5670a/b was non-inferior to bivalent BNT162b2 in terms of immunogenicity, and had a manageable safety profile, when administered as a heterologous booster in children aged 5–11 years.

**Clinical trial registration:**

https://jrct.niph.go.jp/, identifier jRCT2031220665

## 1 Introduction

Data collected during the initial phase of the coronavirus disease 2019 (COVID-19) pandemic suggested that children were less severely affected, with generally milder symptoms and lower mortality rates (1, 2). However, hospitalization rates were disproportionately high among children and adolescents during the omicron wave (3, 4), with some patients experiencing multisystem inflammatory syndrome in children (MIS-C) and long-term sequelae such as cardiac abnormalities (5, 6). Children have also been proposed to be a major reservoir for transmission within households and communities, particularly as new variants emerge (7–9).

Globally, adults were initially prioritized for vaccination, due to their greater risk of severe symptoms and higher mortality rates (10). Vaccination for children aged 5–11 years with messenger ribonucleic acid (mRNA)-based vaccine BNT162b2 (Comirnaty, Pfizer-BioNTech) was first approved in the US and Europe in 2021 (11, 12). Use of bivalent mRNA vaccines in this age group followed in 2022, and as of 2023/24, new monovalent vaccines which include spike protein mRNA from the XBB.1.5 sub-lineage are in use (13). Overall, vaccination in children aged 5–11 years has been shown to be safe and effective, with reduced rates of COVID-19 symptoms, COVID-related hospitalizations, and MIS-C (11, 12, 14–17). Vaccination among children has also been shown to prevent onward transmission of severe acute respiratory syndrome-coronavirus-2 (SARS-CoV-2) (8).

DS-5670 is an mRNA vaccine platform for COVID-19 prophylaxis developed by Daiichi Sankyo Co., Ltd. (Tokyo, Japan). The monovalent vaccine DS-5670a, which incorporated mRNA encoding the receptor-binding domain (RBD) of the spike protein derived from the original Wuhan strain of SARS-CoV-2 (18), was found to provide favorable immune responses along with a clinically acceptable safety profile (19) and was subsequently authorized in Japan for booster immunization against COVID-19. Non-inferiority studies of monovalent DS-5670a and bivalent DS-5670a/b (containing RBD antigens from the original strain and the omicron BA.4-5 variant) as booster vaccinations in adults found that both versions were safe and well-tolerated, and were highly effective against symptomatic COVID-19 with broad neutralization activity across omicron sub-lineages, compared with other authorized LNP-mRNA vaccines (Daiichi Sankyo Co., Ltd., data on file).

Herein, we report data from a phase 2/3 non-inferiority study conducted in children aged 5–11 years who had completed a primary immunization series with monovalent BNT162b2 (against the original strain), which aimed to evaluate the safety and immunogenicity of a third booster dose of DS-5670a/b in this age group.

## 2 Materials and methods

### 2.1 Study design, interventions, and blinding

This was a phase 2/3 study conducted at 42 sites across Japan (**Supplementary Table 1**), and was registered with the Japan Registry of Clinical Trials with the identifier jRCT2031220665. The study was conducted in accordance with the Declaration of Helsinki, Good Clinical Practice guidelines, and all national and regional ordinance, and was approved by the relevant ethical committees at each study site. Written informed consent was obtained from the legal representative of each participant prior to study vaccine administration; wherever possible, participants were also provided with age-appropriate information and asked for their consent.

The design of the study is shown in **Supplementary Figure 1**. Part 1 was an open-label, dose-escalation, sentinel study to assess the safety and tolerability and determine the optimal dose of DS-5670a/b (10 µg or 20 µg mRNA) as a third booster for children aged 5–11 years who had completed a primary immunization series with an approved SARS-CoV-2 vaccine. The safety of DS5670a/b was evaluated to determine the optimal dose for the non-inferiority Part 2. An Advisory Board evaluated and reviewed adverse event (AE) data reported up to 72 hours after administration of DS-5670a/b 10 μg and confirmed that there were no safety concerns before starting administration of DS-5670a/b 20 μg. AE data from participants in the DS-5670a/b 20 μg cohort up to 72 hours after administration were likewise evaluated and reviewed by the Advisory Board, and the study moved to Part 2 after confirmation that there were no safety concerns. Criteria for suspension were occurrence of a serious AE in at least one participant per cohort, or more than one severe AE per participant in at least two members of a cohort. Based on the safety data produced during Part 1, a dose of 20 µg of mRNA was selected for DS-5670a/b for Part 2. Part 2 was a multicenter, randomized, active-controlled, observer-blinded, non-inferiority study, in which participants were randomly assigned in a 1:1 ratio to receive either DS-5670a/b (20 µg mRNA) or bivalent BNT162b2 original/omicron BA.4-5 (10 µg of mRNA). Assignment was stratified by study site and history of SARS-CoV-2 infection. An independent statistician prepared a random assignment table to map participants to the study vaccine groups, and unblinded site personnel were responsible for dispensation and administration of the study vaccine. Investigators were blinded to study vaccine assignment, and uploaded participant data via an interactive web response system. The participants and their legal representatives, the study monitor and members of the study advisory board, sponsor and collaborators, and personnel performing antibody titer determination were also blinded to study vaccine assignment. The first participant enrolment occurred on 27 May 2023, and the observation/follow-up period for Part 2 was planned to continue for 52 weeks post-administration. For this analysis, a cut-off date of 2 December 2023 was applied, at which time all enrolled patients had efficacy data up to Day 29 for analysis of the primary efficacy endpoint.

DS-5670a/b or bivalent BNT162b2 were administered intramuscularly into the deltoid region of the upper arm on Day 1. Although both DS-5670 and BNT162b2 are both classified as LNP-mRNA vaccines, and the basic mechanism of action of each is similar (i.e., delivery and release of the mRNA antigen into the cytosol of immune cells), the precise compositions of the two vaccines differ. As previously reported, the antigen component of BNT162b2 comprises the full-length spike protein from SARS-CoV-2 and that of DS-5670 encompasses only the RBD (19), while the lipid composition of the LNP component is proprietary to each manufacturer. The comparator bivalent BNT162b2 vaccine for ages 5–11 years was supplied by the Japanese government to be used as the study control.

### 2.2 Participants

This study included children (aged 5–11 years) who had fully completed the two-dose primary vaccination series with monovalent BNT162b2 vaccine against the original SARS-CoV-2 strain at least 3 months prior to enrolment. Key exclusion criteria were presence of serious cardiovascular disease or current/previous myocarditis or pericarditis; any kidney, liver, hematologic (including thrombocytopenia or coagulation disorder), neuropsychiatric, or developmental disorder; history of seizures or epilepsy due to vaccination, or history of anaphylaxis or severe allergy due to administration of food, drugs, cosmetics or vaccines; or a previous diagnosis of immunodeficiency or a close relative with congenital immunodeficiency. Children with symptoms suggestive of SARS-CoV-2 infection or close contacts of infected persons at the time of obtaining informed consent, or a diagnosis of SARS-CoV-2 infection by reverse transcriptase-polymerase chain reaction testing, or a positive SARS-CoV-2 antigen test at enrolment were also ineligible for participation. However, children who had recovered from COVID-19 at least 3 months prior to enrolment were eligible for study inclusion.

### 2.3 Assessments and outcomes

The study objective was to demonstrate the non-inferiority of DS-5670a/b to bivalent BNT162b2, in terms of immunogenicity at Day 29 after the third booster administration, and to evaluate safety.

The primary efficacy endpoint was blood neutralizing activity geometric mean titer (GMT) against SARS-CoV-2 (omicron variant BA.5.2.1) and immune response rate on Day 29 after the third booster administration. The immune response rate was defined as the proportion of participants with a ≥ 4-fold increase in post-vaccination circulating anti-SARS-CoV-2 neutralizing activity relative to pre-vaccination levels. Other efficacy endpoints were blood neutralizing activity GMT against SARS-CoV-2 (original strain, BA.5.2.1, BQ.1.1.3, and XBB.1.5.6) and immune response rate on Day 29, and the incidence of COVID-19 up to 52 weeks after booster administration. Variants were selected according to those circulating most widely in Japan at the time the study was planned and conducted (20). Safety endpoints included the occurrence of solicited AEs, unsolicited AEs, and serious AEs (SAEs), and clinically relevant changes in laboratory values.

Blood samples for immunogenicity and laboratory (safety and COVID-19) testing were collected pre-administration, and at 4, 12, 26, and 52 weeks after administration of the study drug or at the time of study discontinuation. A total of 11 mL of blood was collected on Days 1 and 29, and 5 mL at each time point thereafter.

Neutralizing titers were assessed *in vitro* using the original SARS-CoV-2 strain (2019-nCoV/Italy-INMI1) and the variants of interest for this study (BA.5.2.1, BQ.1.1.3 and XBB.1.5.6; live virus obtained from the Rega Institute Leuven, Belgium) based on the cytopathic effect in cultured cells using the methodology previously reported (19). Briefly, heat-inactivated (approximately 30 min at 56°C) serum samples were incubated with the virus suspension for 1 hour, then added to VeroE6 cells and incubated (37°C and 5% CO_2_) for either 3 days (original strain) or 4 days (omicron variants). An inverted light microscope was used to evaluate the cytopathic effect; the reciprocal of the highest dilutions that protected at least 50% of cells from the cytopathic effect was designated as the neutralization titer. Titers for the original strain were converted to International Units using the published international standard (21).

The legal representative recorded participant health status in an electronic diary from Day 1 until 4 weeks after administration of the study drug. Solicited AEs (injection site events [redness, swelling, induration, pain, warmth, and pruritus] and systemic events [fever, malaise, headache, rash, and myalgia]) were collected up to 7 days post-administration, unsolicited AEs up to 28 days, and SAEs from the time of informed consent to 52 weeks. Laboratory tests were performed pre-administration and at 28 days post-administration. AEs were coded using the Medical Dictionary for Regulatory Activities version 26.1.

### 2.4 Statistical analysis

To compare vaccine boosters, the ratio of neutralizing activity against SARS-CoV-2 (omicron variant BA.5.2.1) in the blood on Day 29 was calculated for DS-5670a/b versus bivalent BNT162b2. To confirm non-inferiority the lower limit of the two-sided 95% confidence interval (CI) of the GMT ratio was required to be above the non-inferiority margin of 0.67, and the lower limit of the two-sided 95% CI for the difference in the immune response rate was required to exceed −10% in favor of DS-5670a/b. Assuming that the true GMT ratio (DS-5670a/b group versus bivalent BNT162b2 group) for blood neutralizing activity against SARS-CoV-2 at Day 29 after study vaccine administration was 1.10, and the common standard deviation (SD) of blood neutralizing activity (common logarithmic value) was 0.490, the true immune response rate was estimated to be 97.5% for both groups with an one-sided alpha of 2.5%. After taking into account anticipated dropout rates, the number of participants required to power the immunogenicity non-inferiority comparison was 204 (of whom 102 would receive DS-5670a/b and 102 would receive bivalent BNT162b2).

The primary analysis population for immunogenicity was the immunogenicity-evaluable per protocol set (PPS), which included participants who received a dose of study drug, had a pre-administration and at least one post-administration immunogenicity measurement, and had no protocol violations that could affect the immunogenicity evaluations. The analysis population for COVID-19 incidence was the efficacy-evaluable PPS, which included participants who completed protocol-specified study procedures and had no significant protocol violations that could affect the efficacy evaluation. Immunogenicity and efficacy analyses were performed based on the study vaccine group to which each participant was assigned. AEs were evaluated in the safety analysis set, which included all participants who underwent study vaccine administration; safety analyses were performed based on the study drug that was actually administered. Solicited AEs were evaluated in the solicited safety analysis set, which included all participants in the safety analysis set for whom information on the occurrence of at least one solicited AE was available.

Baseline participant characteristics and safety data were recorded descriptively as number (%), mean (SD), or median (range). The adjusted GMT of blood neutralizing activity was calculated with a two-sided 95% CI. A linear model was applied with common log transformed neutralizing titers as the dependent variable, the study vaccine group as the independent variable, and the common log transformed baseline titer and presence or absence of a history of SARS-CoV-2 infection as covariates. The adjusted between-group difference in the immune response rate, along with a two-sided 95% CI, was also calculated. The Mantel-Haenszel method was applied, and presence or absence of a history of SARS-CoV-2 infection included as a stratum. The COVID-19 incidence rate was calculated as the number of cases per 1000 person-years. All statistical calculations were performed using SAS software version 9.4 or later (SAS Institute, Inc., Cary, NC, USA).

## 3 Results

### 3.1 Study part 1

#### 3.1.1 Dose selection for part 2

Four participants were assigned to the DS5670a/b 10 μg cohort and three to the DS5670a/b 20 μg cohort. Among the DS5670a/b 10 μg cohort, there were no serious or severe solicited or unsolicited AEs. However, one had a moderate solicited systemic AE of malaise, which resolved on the same day. No further safety concerns were noted. Three participants subsequently received DS5670a/b 20 μg, of whom one had solicited AEs of moderate grade malaise and severe grade fever, and an unsolicited AE of moderate grade influenza. These resolved within a week of onset. Since the influenza was judged to be unrelated to the DS-5670a/b vaccination, and the malaise and fever were likely to be related to the influenza, the Advisory Board considered that Part 2 of the study could commence, with the optimal DS-5670a/b dose determined to be 20 μg.

### 3.2 Study part 2

#### 3.2.1 Study population

**Figure 1:**
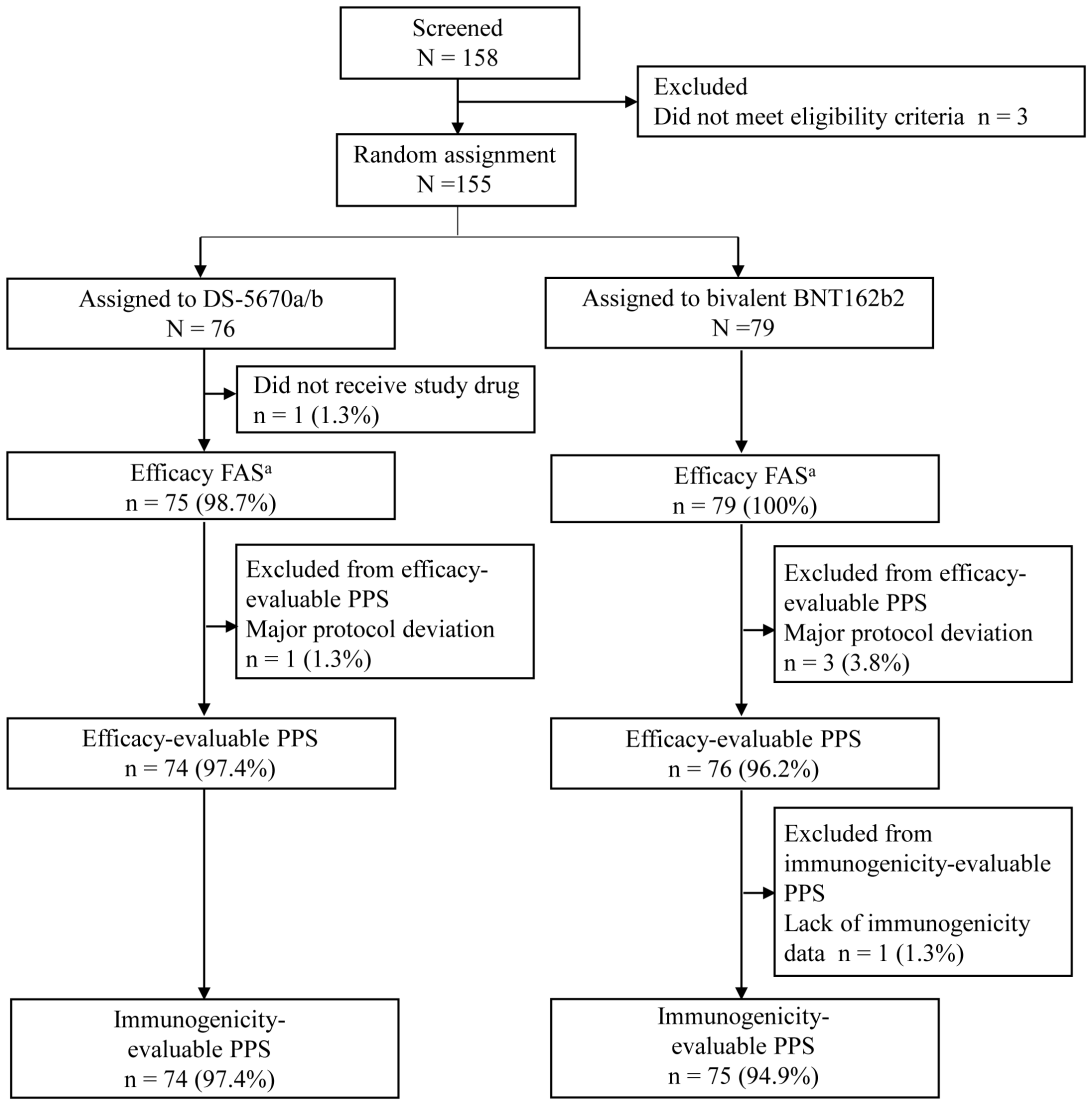
CONSORT diagram of participant disposition and analysis populations ^a^The efficacy FAS was identical to the safety analysis set. FAS, full analysis set; PPS, per protocol set.

A total of 155 participants were randomly assigned to receive study vaccination in Part 2, of whom 154 (DS-5670a/b, n = 75; bivalent BNT162b2, n = 79) received the third booster vaccination. Data collected up to Day 29 are included in this report, and the disposition and analysis populations used for the calculation of results are presented in **Figure 1**. After exclusions due to major protocol deviations, the efficacy-evaluable PPS consisted of 74 participants in the DS-5670a/b group and 76 in the bivalent BNT162b2 group. One participant was excluded from immunogenicity evaluation because they did not have an immunogenicity measurement on the Day 29 visit; thus, the immunogenicity-evaluable PPS included 74 participants in the DS-5670a/b group and 75 in the bivalent BNT162b2 group.

The demographics and baseline characteristics of participants are described in **Table 1**. The mean age of participants was 9.0 years (range 5 to 11 years), and approximately half (73/149, 49.0%) were male. The majority of participants (141/149, 94.6%) had a vaccine interval since completion of their second monovalent BNT162b2 injection of ≥ 6 months (median interval 13.8 months [range 3.9 to 18.8 months]). Almost half of participants (71/149, 47.0%) reported a prior history of SARS-CoV-2 infection, with almost all being self-reported. Characteristics were generally similar across the vaccination groups, and there were no notable differences between the immunogenicity-evaluable PPS and the safety analysis set.

**Table 1:** Baseline demographics and clinical characteristics (immunogenicity-evaluable PPS).

	DS-5670a/b (n = 74)	Bivalent BNT162b2 (n = 75)	All participants (N = 149)
Age (years), median (range)	9.0 (5, 11)	9.0 (5, 11)	9.0 (5, 11)
Male sex, n (%)	35 (47.3)	38 (50.7)	73 (49.0)
Weight (kg), median (range)	30.3 (13.2, 76.9)	28.8 (17.7, 63.9)	29.5 (13.2, 76.9)
Height (cm), median (range)	132.7 (105.5, 158.2)	132.0 (108.4, 157.3)	132.5 (105.5, 158.2)
Vaccination interval since second dose of monovalent BNT162b2			
Duration (months), median (range)	13.7 (3.9, 18.7)	14.1 (4.7, 18.8)	13.8 (3.9, 18.8)
Patients with interval 3 to < 6 months, n (%)	6 (8.1)	2 (2.7)	8 (5.4)
Patients with interval ≥ 6 months, n (%)	68 (91.9)	73 (97.3)	141 (94.6)
History of SARS-CoV-2 infection, n (%)	35 (47.3)	36 (48.0)	71 (47.7)
Self-reported prior infection	35 (47.3)	35 (46.7)	70 (47.0)
Positive antibody test^a^	2 (2.7)	3 (4.0)	5 (3.4)

PPS, per protocol set; SARS-CoV-2, severe acute respiratory syndrome-coronavirus-2.

All participants (100%) were of Asian race, and all had received two prior monovalent BNT162b2 (original strain) vaccinations.

a N-antibody positivity was confirmed by an immunochromatographic test kit (Rapidfields S+N IgG [RF-NC003]; Kurabo Industries Ltd., Osaka, Japan).

#### 3.2.2 Immunogenicity/effectiveness

A summary of serum neutralization titers against SARS-CoV-2 (omicron variant BA.5.2.1) is described in **Table 2**. The adjusted GMT ratio of DS-5670a/b to bivalent BNT162b2 on Day 29 was 1.636 (95% CI, 1.221, 2.190). Immune response rates were ≥ 89% in both study vaccine groups; in a pairwise comparison, the adjusted difference between DS-5670a/b and bivalent BNT162b2 was 2.6% (95% CI, –7.8, 13.8). Both of these results exceeded the prespecified non-inferiority margins, and the study met the primary endpoint.

**Table 2:** Summary of neutralizing antibody titers and immune response rates against SARS-CoV-2 omicron variant BA.5.2.1 (immunogenicity-evaluable PPS).

	DS-5670a/b (n = 74)	Bivalent BNT162b2 (n = 75)
Baseline (Day 1, predose)		
Participants with evaluable data, n	73	75
Neutralizing antibody titer		
GMT (95% CI)^a^	72.421 (50.366, 104.134)	79.274 (53.783, 118.845)
Day 29		
Participants with evaluable data, n	67	59
Neutralizing antibody titer		
GMT (95% CI)^a^	1692.483 (1310.447, 2185.893)	965.504 (729.071, 1278.609)
Adjusted GMT (95% CI)^b^	1644.228 (1346.417, 2007.912)	1005.274 (812.690, 1243.494)
Adjusted GMT ratio (95% CI)^b^	1.636 (1.221, 2.190)	
GMFR (95% CI)^a^	26.421 (19.534, 35.736)	18.098 (13.184, 24.843)
Immune response rate, % (95% CI)^c^	92.5 (83.4, 97.5)	89.8 (79.2, 96.2)
Adjusted difference, % (95% CI)^d^	2.6 (–7.8, 13.8)	
Non-inferiority criteria	Met	

ANCOVA, analysis of covariance; CI, confidence interval; GMFR, geometric mean fold rise; GMT, geometric mean titer; PPS, per protocol set; SARS-CoV-2, severe acute respiratory syndrome-coronavirus-2.

Ratios were defined as DS-5670a/b/bivalent BNT162b2.

a 95% CI was calculated based on the Student’s t-distribution of the log-transformed values or the difference in the log-transformed values for GMT and GMFR, respectively, then back transformed to the original scale for presentation.

b The adjusted GMT and its 95% CI was calculated based on a linear ANCOVA model with common log transformed neutralizing titers as the dependent variable, the study vaccine group as the independent variable, and the common log transformed baseline titer and presence or absence of a history of SARS-CoV-2 infection as covariates.

c 95% CI was calculated using the Clopper Pearson exact method.

d The adjusted response rate difference was calculated by Mantel-Haenszel methods and its 95% CI calculated based on stratified Wilson-Newcombe score methods.

GMT and immune response rates against BA.5.2.1 according to the presence or absence of historical SARS-CoV-2 infection are reported in **Supplementary Table 2**. As expected, study participants with a history of infection had higher Day 1 antibody titers than those without prior infection. However, both sub-populations showed high GMTs and immune response rates at Day 29 in response to study vaccination, indicating good booster immunogenicity regardless of infection history.

**Figure 2:**
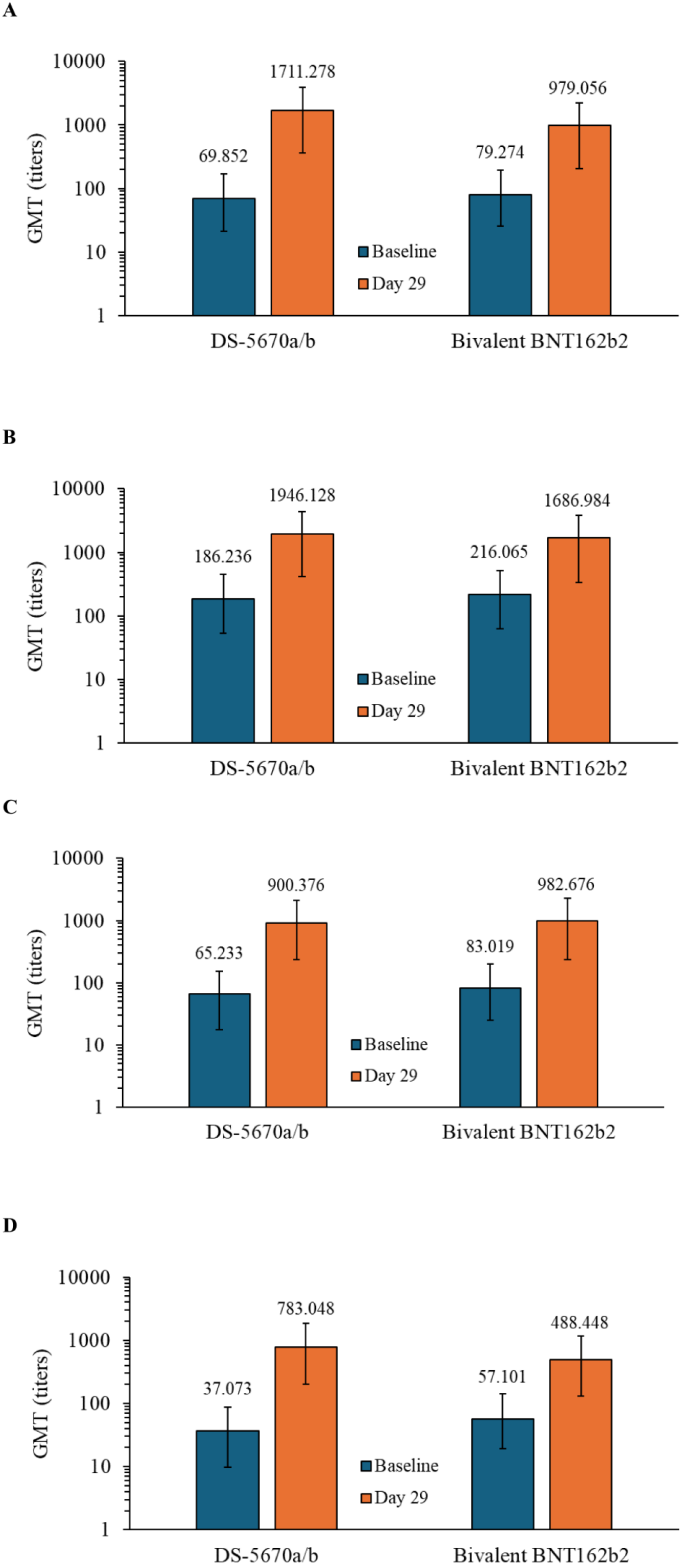
Immunogenicity at baseline and on Day 29. GMTs against the **(A)** omicron BA.5.2.1 strain, **(B)** SARS-CoV-2 original strain, **(C)** BQ.1.1.3 strain, and **(D)** XBB.1.5.6 strain (immunogenicity-evaluable PPS). DS-5670a/b and BNT162b2 both contain antigens against the original and omicron BA.4-5 strains. Vertical bars represent 95% confidence intervals. GMT, geometric mean titer; PPS, per protocol set.

Serum neutralization titers against SARS-CoV-2 variants including the original, BA.5.2.1, BQ.1.1.3, and XBB.1.5.6 strains at baseline (Day 1, predose) and on Day 29 are shown in **Figure 2**. DS-5670a/b induced neutralization titers against each strain evaluated, at comparable or greater levels than bivalent BNT162b2, indicating broad neutralization activity across recent omicron sublineages.

No cases of COVID-19 (based on positive SARS-CoV-2 infection test and any COVID-19 symptom) were reported between Day 8 and Day 29 post-administration in either study vaccine group. Although this was not long enough to confirm long-term infection prevention, it demonstrated comparable short-term effectiveness for DS-5670a/b and bivalent BNT162b2 in preventing infection in this age group.

#### 3.2.3 Safety

AEs occurring during the study are summarized in **Table 3**. The majority of participants experienced at least one AE (DS-5670a/b, 66/75 [88.0%]; bivalent BNT162b2, 72/79 [91.1%]). There were no serious AEs, no AEs leading to study discontinuation, and no fatal AEs in either group. Severe solicited AEs in the DS-5670a/b group included swelling (n = 1) and warmth (n = 1) at the injection site, and fever (n = 3). All resolved within 2 days of onset. One participant in the bivalent BNT162b2 group had severe fever and severe malaise; the fever resolved within 1 day and the malaise after 11 days.

**Table 3:** Summary of AEs (safety analysis set).

AEs, n (%)	DS-5670a/b (n = 75)	Bivalent BNT162b2 (n = 79)
Participants with at least 1 solicited AE	66 (88.0)	72 (91.1)
Participants with at least 1 severe solicited AE	5 (6.7)	1 (1.3)
Participants with at least 1 solicited injection site AE	65 (86.7)	70 (88.6)
Participants with at least 1 severe solicited injection site AE	2 (2.7)	0
Participants with at least 1 solicited systemic AE	28 (37.3)	23 (29.1)
Participants with at least 1 severe solicited systemic AE	3 (4.0)	1 (1.3)
Participants with at least 1 unsolicited AE	36 (48.0)	33 (41.8)
Participants with at least 1 drug-related unsolicited AE	8 (10.7)	8 (10.1)
Participants with at least 1 severe unsolicited AE	0	0
Participants with at least 1 serious AE	0	0
Participants with at least 1 AE resulting in vaccination/study discontinuation	0	0
Participants with AE leading to death	0	0

AE, adverse event.

Participants with multiple AEs within a category were counted only once for that category. AEs include solicited and unsolicited AEs. Solicited AEs were collected from the start of the study drug until 7 days after the booster administration. Unsolicited AEs were collected from the start of the study drug until 28 days after the booster administration.

**Figure 3:**
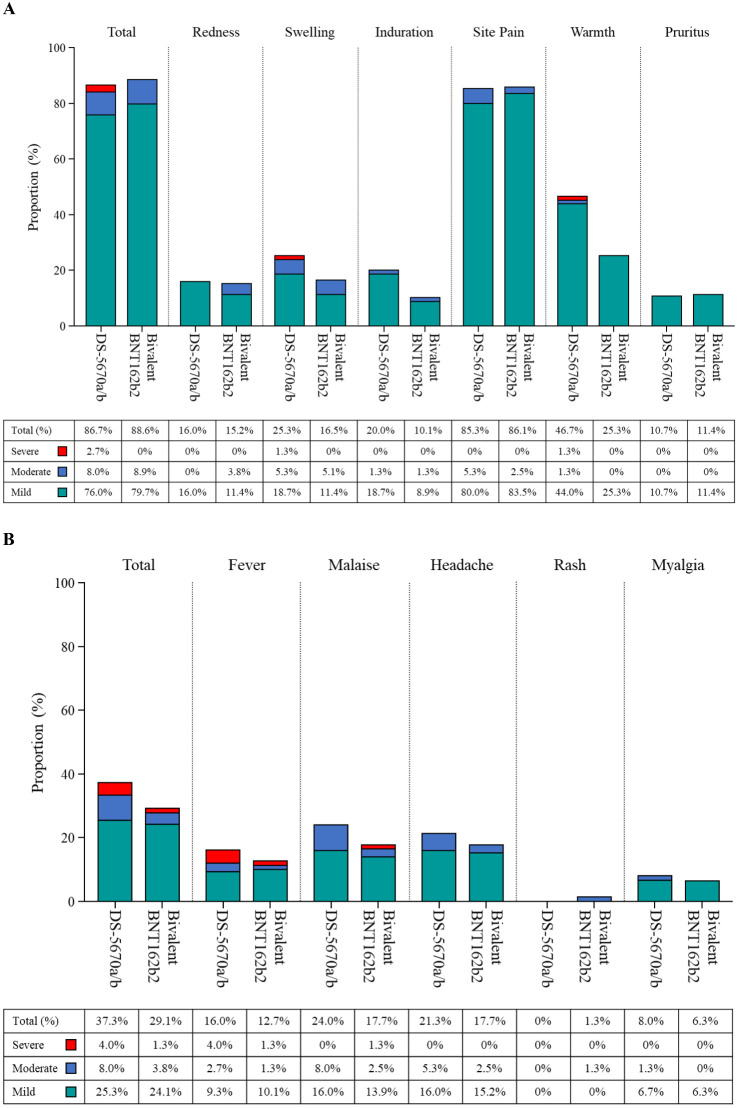
Solicited AEs (safety analysis set). **(A)** injection site AEs; **(B)** systemic AEs. AE, adverse event.

Solicited AEs are shown in **Figure 3**. The majority were mild in intensity. The most common injection site AEs in both vaccination groups were pain and warmth. The most frequently reported solicited injection site AE was mild pain (DS-5670a/b, 80.0% and bivalent BNT162b2, 83.5%). There were no apparent differences in the overall number of injection site AEs or the incidence of each event between study vaccine groups, with the exception of injection site warmth which was reported at a higher rate in the DS-5670a/b group (DS-5670a/b, 46.7% and bivalent BNT162b, 25.3%). Although the overall incidence of solicited systemic AEs was slightly higher in the DS-5670a/b group than in the BNT162b2 group, there was no apparent difference in the incidence of each event between the groups. Solicited systemic AEs that were relatively common in both vaccination groups were malaise and headache.

Unsolicited AEs are described in **Supplementary Table 3**. Injection site events (erythema, pruritus, and swelling) were more common in the DS-5670a/b group, while nasopharyngitis was more common in the bivalent BNT162b2 group.

## 4 Discussion

In this study, the non-inferiority of bivalent DS-5670a/b to bivalent BNT162b2 in terms of neutralizing activity against SARS-CoV-2 (omicron variant BA.5.2.1) at Day 29 after the third booster administration was confirmed in children aged 5-11 years. DS-5670a/b demonstrated high anti-SARS-CoV-2 BA.5.2.1 serum neutralization titers and an immune response rate of 92.5% at Day 29 after booster immunization. The adjusted GMT ratio of 1.636 (95% CI, 1.221, 2.190) and broad cross-neutralization activity against SARS-CoV-2 omicron variants observed in this study were also consistent with data obtained from a study of bivalent DS-5670a/b as a booster (dose 4+) in Japanese adults (Daiichi Sankyo Co., Ltd., data on file).

Data from the current study also showed that booster vaccination with DS-5670a/b was able to protect children against COVID-19, with no reported infections occurring between Day 8 and Day 29. In context, according to data from the Japan Ministry of Health, Labour and Welfare for the period of 22 May 2023 to 3 December 2023 (which approximates to the period during which this study was conducted), the average incidence of COVID-19 among children (aged 0–14 years) was 13,487 per week, which equates to around 30% of the average 44,562 weekly cases reported during this period among all age groups (22). Considering the study size and the limited duration of follow-up at this analysis, it is too early to determine whether the fact that no cases of COVID-19 were reported in our study was a result of the booster vaccination, and the long-term preventive effects of a DS-5670a/b booster dose in children remain to be established. Nonetheless, the non-inferior immunogenicity of DS-5670a/b to the comparator vaccine, which has been shown to prevent development of COVID-19, was confirmed. Therefore, DS-5670a/b is expected to have a prophylactic effect against infection. Such protection may help in reducing the risks of hospitalization and the development of severe symptoms such as MIS-C in the pediatric population (23). Given the ongoing nature of the COVID-19 outbreak, and the continuing emergence of new variants, increasing the number of available vaccine options is an important tactic to maintain public health and decrease the burden on both society and the healthcare system. A recent analysis of US data obtained during the period when the omicron BA.4-5 sublineages were predominant found that bivalent mRNA vaccines provided 54.0% effectiveness against SARS-CoV-2 infection in children, and that infection was less likely compared with those who had received only monovalent vaccination (24). Moreover, studies have suggested that heterologous mRNA-mediated booster vaccinations provide greater benefits, including higher antibody levels and reduced incidence of breakthrough infections, compared with homologous boosters (25–27). Based on the results of the current study, heterologous booster vaccination with DS-5670a/b was able to provide high levels of pediatric immunogenicity across several omicron sublineages. The DS-5670 LNP-mRNA vaccine platform has shown effectiveness against SARS-CoV-2 strains circulating during the pandemic when formulated as either monovalent or bivalent composition, and with differing RBD antigens derived from the original strain or variants [Daiichi Sankyo Co., Ltd., data on file and (19)]. As such, the manufacture of new DS-5670 compositions, targeting emergent strains, should allow a rapid and effective response against future variants of concern, thereby providing ongoing protection for vulnerable individuals of all ages.

The results of the safety analysis indicated that DS-5670a/b was safe to use in this age group, with an adverse event profile similar to that of the comparator, and there were no novel concerns in the pediatric population at the time of data cut-off. To date, no events of myocarditis or pericarditis have been reported following administration of any DS-5670-based vaccine, and no serious vaccine-related AEs have occurred.

The limitations of this study include the geographic restriction to Japan, which may limit the generalizability of the data, and the short duration of follow-up, which precludes any inferences regarding enduring protection against COVID-19 or long-term safety in the study population. In addition, although an antibody test for SARS-CoV-2 infection was performed at each visit, and individuals with a negative antibody test on Day 1 and a positive antibody result on Day 29 were excluded from the primary analysis, we cannot definitively state that all asymptomatic cases were eliminated from the immunogenicity evaluable population. Thus, there is a small chance that hybrid immunity may have affected the immunogenicity measures, although the potential risk of this bias was equal among both study vaccine groups.

In summary, based on Day 29 post-vaccination assessments, the bivalent vaccine DS-5670a/b provided non-inferior immunogenicity to SARS-CoV-2 compared with bivalent BNT162b2, protected against COVID-19 infection, and had a manageable safety profile, when administered as a heterologous booster in children aged 5–11 years.

## Data Availability

The raw data supporting the conclusions of this article will be made available by the authors, without undue reservation.
