# Supplementary material for: Booster vaccination using bivalent DS-5670a/b is safe and immunogenic against SARS-CoV-2 variants in children aged 5–11 years: a phase 2/3, randomized, active-controlled study
Source: Front Immunol. 2024 Sep 2;15:1445459. doi: 10.3389/fimmu.2024.1445459 (PMC11403248; doi:10.3389/fimmu.2024.1445459)
Supplement: Supplementary file 1 [file Datasheet1.docx]

Supplementary Material

**Supplementary Table 1.** Listing of study sites and investigators

| **Study site** | **Investigator** |
| --- | --- |
| Aizenbashi Hospital | Masashi Shiomi |
| Clinical Research Hospital Tokyo | Hiroyuki Fukase |
| Fujimaki Ent Clinic | Yutaka Fujimaki |
| Fukuwa Clinic | Yasushi Fukushima |
| Funai Ent Clinic | Shigehiro Ueyama |
| Hakodate Central General Hospital | Takeshi Kida |
| Hidaka Children's Clinic | Hidenobu Hidaka |
| Himawarikai Kanagawa Himawari Clinic | Ryuta Ono |
| Hoshinaga Otolaryngology Clinic | Keisuke Hoshinaga |
| Ikeda Naika Clinic | Motohisa Ikeda |
| Iwaya Children's Clinic | Minako Iwaya |
| Kagoshima Children's Hospital | Noboru Uchikado |
| Kaiseikai Kita Shin Yokohama Internal Medicine Clinic | Uguri Kamiya |
| Kakurenbo Clinic of Pediatrics and Pediatric Allergy | Chizuru Okada |
| Kawagoe Otology Institute | Hideaki Sakata |
| Kawasaki Municipal Hospital | Takatoshi Tsuchihashi |
| Kunitachi Sakura Hospital | Kiyoshi Nakamura |
| Matsuo Kenko Clinic | Kaneyuki Matsuo |
| Medical Corporation Isenkai Izumigaoka Skin and Endoscopy Clinic | Masaru Hitomi |
| Medical Corporation Kanyukai Kikumori Otolaryngology Clinic | Hiroshi Kikumori |
| Medical Corporation Shinanokai, Shinanozaka Clinic | Kenji Takazawa |
| MIH Clinic Yoyogi | Toshio Miyata |
| Miyake Clinic | Shuji Miyake |
| National Hospital Organization Kanazawa Medical Center | Kazuhide Ohta |
| National Hospital Organization Nagara Medical Center | Michinori Funato |
| Nijiiro Pediatric Clinic | Kei Takemoto |
| Niwa Family Clinic | Kiyoshi Niwa |
| Public Health Insurance Association Clinic | Shinichiro Hosokawa |
| Rinku General Medical Center | Masaya Yamato |
| Seijunkai Mito Hospital | Hiromitsu Tsuchida |
| Shibuya Clinic | Tomoyuki Shibuya |
| Shimamura Memorial Hospital | Kentaro Masuko |
| Shindo Children's Clinic | Shizuo Shindo |
| Shizuoka Welfare Hospital | Toshihiro Tanaka |
| Suzuran Children's Clinic | Keisaku Imamura |
| Takasaki Pediatrics Clinic | Yoshio Takasaki |
| Tenjin Sogo Clinic | Kenjiro Nakamura |
| Tokyo-Eki Center-building Clinic | Arihiro Kiyosue |
| Ueyama Child Clinic | Nami Ueyama |
| Yamasaki Family Clinic | Koichiro Yamasaki |
| Yamashita Pediatrics Clinic | Hisashi Yamashita |
| Yoshimura Child Clinic | Ryota Yoshimura |

**Supplementary Table 2.** Summary of neutralizing antibody titers and immune response rates against SARS-CoV-2 omicron variant BA.5.2.1 according to presence or absence of historical SARS-CoV-2 infection (immunogenicity-evaluable PPS)

|  | **DS-5670a/b**  **(n = 74)** | **Bivalent BNT162b2**  **(n = 75)** |
| --- | --- | --- |
| **Presence of historical SARS-CoV-2 infection** |  |  |
| **Baseline (Day 1, predose)** |  |  |
| Participants with evaluable data, n | 35 | 36 |
| Neutralizing antibody titer |  |  |
| GMT (95% CI)^a^ | 126.169 (84.680, 187.988 | 145.298 (93.551, 225.668) |
| **Day 29** |  |  |
| Participants with evaluable data, n | 32 | 29 |
| Neutralizing antibody titer |  |  |
| GMT (95% CI)^a^ | 2425.052  (1896.071, 3101.614) | 1425.347  (1009.057, 2013.379) |
| GMFR (95% CI)^a^ | 21.202 (14.365, 31.291) | 12.752 (8.220, 19.783) |
| Immune response rate, % (95% CI)^b^ | 93.8 (79.2, 99.2) | 82.8 (62.4, 94.2) |
| **Absence of historical SARS-CoV-2 infection** |  | |
| **Baseline (Day 1, predose)** |  |  |
| Participants with evaluable data, n | 38 | 39 |
| Neutralizing antibody titer |  |  |
| GMT (95% CI)^a^ | 43.432 (24.775, 76.138) | 45.315 (25.129, 81.714) |
| **Day 29** |  |  |
| Participants with evaluable data, n | 35 | 30 |
| Neutralizing antibody titer |  |  |
| GMT (95% CI)^a^ | 1218.191  (802.365, 1849.521) | 662.561  (437.983, 1002.294) |
| GMFR (95% CI)^a^ | 32.310 (20.313, 51.393) | 25.387 (16.251, 39.660) |
| Immune response rate, % (95% CI)^b^ | 91.4 (76.9, 98.2) | 96.7 (82.8, 99.9) |

ANCOVA, analysis of covariance; CI, confidence interval; GMFR, geometric mean fold rise; GMT, geometric mean titer; PPS, per protocol set; SARS-CoV-2, severe acute respiratory syndrome-coronavirus-2.

^a^95% CI was calculated based on the Student's t-distribution of the log-transformed values or the difference in the log-transformed values for GMT and GMFR, respectively, then back transformed to the original scale for presentation.

^b^95% CI was calculated using the Clopper Pearson exact method.

**Supplementary Table 3.** Unsolicited AEs occurring in >1 participant in either group in Part 2 (safety analysis set)

| **MedDRA Preferred Term, n (%)** | **DS-5670a/b**  **(n = 75)** | **Bivalent BNT162b2**  **(n = 79)** |
| --- | --- | --- |
| Injection site erythema | 8 (10.7) | 1 (1.3) |
| Injection site pruritus | 6 (8.0) | 1 (1.3) |
| Injection site swelling | 5 (6.7) | 0 |
| Pharyngitis | 4 (5.3) | 3 (3.8) |
| Nasopharyngitis | 3 (4.0) | 11 (13.9) |
| Cough | 3 (4.0) | 2 (2.5) |
| Influenza | 2 (2.7) | 2 (2.5) |
| Gastroenteritis | 2 (2.7) | 1 (1.3) |
| Diarrhea | 2 (2.7) | 0 |
| Oropharyngeal pain | 2 (2.7) | 0 |
| Pyrexia | 1 (1.3) | 3 (3.8) |
| Headache | 1 (1.3) | 2 (2.5) |
| Pruritus | 0 | 2 (2.5) |
| Vomiting | 0 | 2 (2.5) |

AE, adverse event; MedDRA, Medical Dictionary for Regulatory Activities.

**Supplementary Figure 1.** Study design

**
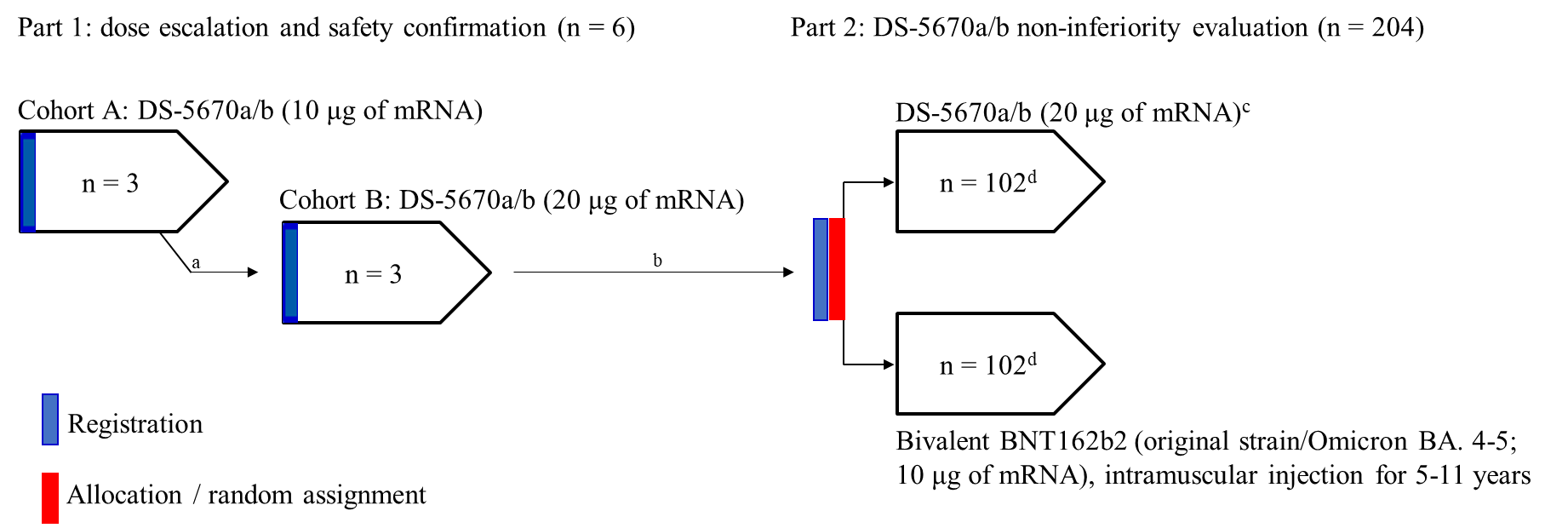
**

^a^Safety up to 72 hours after administration of DS-5670a/b was evaluated to determine whether or not to proceed to the next cohort.
^b^Safety up to 72 hours after administration of DS-5670a/b was evaluated to determine whether to proceed to Part 2.
^c^Dose was determined based on the results of Part 1.
^d^Planned enrolment.
